# A cluster-randomized trial of mass drug administration with a gametocytocidal drug combination to interrupt malaria transmission in a low endemic area in Tanzania

**DOI:** 10.1186/1475-2875-10-247

**Published:** 2011-08-24

**Authors:** Seif A Shekalaghe, Chris Drakeley, Sven van den Bosch, Roel ter Braak, Wouter van den Bijllaardt, Charles Mwanziva, Salimu Semvua, Alutu Masokoto, Frank Mosha, Karina Teelen, Rob Hermsen, Lucy Okell, Roly Gosling, Robert Sauerwein, Teun Bousema

**Affiliations:** 1 Kilimanjaro Christian Medical College-Kilimanjaro Clinical Research Institute, Moshi, Tanzania; 2 Department of Medical Microbiology, Radboud University Nijmegen Medical Centre, Nijmegen, Netherlands; 3 Ifakara Health Institute, Bagamoyo Research and Training Centre, Bagamoyo, Tanzania; 4 Department of Immunology & Infection; Faculty of Infectious and Tropical Diseases, London School of Hygiene and Tropical Medicine, London, UK; 5 MRC Centre for Outbreak Analysis & Modelling, Department of Infectious Disease Epidemiology, Imperial College London, London, UK

## Abstract

**Background:**

Effective mass drug administration (MDA) with anti-malarial drugs can clear the human infectious reservoir for malaria and thereby interrupt malaria transmission. The likelihood of success of MDA depends on the intensity and seasonality of malaria transmission, the efficacy of the intervention in rapidly clearing all malaria parasite stages and the degree to which symptomatic and asymptomatic parasite carriers participate in the intervention. The impact of MDA with the gametocytocidal drug combination sulphadoxine-pyrimethamine (SP) plus artesunate (AS) plus primaquine (PQ, single dose 0.75 mg/kg) on malaria transmission was determined in an area of very low and seasonal malaria transmission in northern Tanzania.

**Methods:**

In a cluster-randomized trial in four villages in Lower Moshi, Tanzania, eight clusters (1,110 individuals; cluster size 47- 209) were randomized to observed treatment with SP+AS+PQ and eight clusters (2,347 individuals, cluster size 55- 737) to treatment with placebo over three days. Intervention and control clusters were 1km apart; households that were located between clusters were treated as buffer zones where all individuals received SP+AS+PQ but were not selected for the evaluation. Passive case detection was done for the entire cohort and active case detection in 149 children aged 1-10 year from the intervention arm and 143 from the control arm. Four cross-sectional surveys assessed parasite carriage by microscopy and molecular methods during a five-month follow-up period.

**Results:**

The coverage rate in the intervention arm was 93.0% (1,117/1,201). Parasite prevalence by molecular detection methods was 2.2-2.7% prior to the intervention and undetectable during follow-up in both the control and intervention clusters. None of the slides collected during cross-sectional surveys had microscopically detectable parasite densities. Three clinical malaria episodes occurred in the intervention (n = 1) and control clusters (n = 2).

**Conclusions:**

This study illustrates the possibility to achieve high coverage with a three-day intervention but also the difficulty in defining suitable outcome measures to evaluate interventions in areas of very low malaria transmission intensity. The decline in transmission intensity prior to the intervention made it impossible to assess the impact of MDA in the chosen study setting.

**Trial Registration:**

ClinicalTrials.gov: NCT00509015

## Background

Recent reports on a reduction of malaria transmission in several parts of sub-Saharan Africa [1-7] have resulted in optimism about the possibility to eliminate malaria in some malaria endemic areas [8,9]. Significant reductions in the number of clinical malaria cases were attributed to the wide-scale implementation of vector control measures [1,2,10,11], high coverage of insecticide-treated bed nets [1,2,4,6,10,11] and the introduction of effective artemisinin combination therapy (ACT) [1-3,6,12]. ACT has excellent cure rates in sub-Saharan Africa [13] and has an additional advantage of reducing malaria transmission in comparison to previous first-line treatments [14-20]. However, even at very low levels of transmission intensity, the employment of ACT in the first-line treatment of malaria may not result in the elimination of malaria [15]. This is because the impact of treatment on transmission intensity is greatly influenced by the proportion of parasite carriers that carry the infection asymptomatically and that are therefore unlikely to seek anti-malarial treatment [18]. Asymptomatic parasite carriers form an important source of infection to mosquitoes [21-26] and frequently harbour parasites at densities below the microscopic detection limit [14,27-31]. Sub-microscopic parasite carriage may be most relevant for malaria transmission in areas of low endemicity, where the relative proportion of parasite carriers that are missed by microscopy is greatest [30]. These low-density infections should therefore be included in interventions that aim to target the human infectious reservoir.

There are two possible approaches to include asymptomatic carriers in treatment campaigns: i) mass-screening and treatment of all parasite carriers (MSAT) and ii) mass treatment without prior screening for parasitaemia, i.e. mass drug administration (MDA). Large-scale and rapid detection of sub-microscopic parasite densities under field conditions is currently complicated [27] primarily due to the sensitivity and turn-around time of the tests available. An MDA approach may, therefore, be the most straightforward method. Several MDA interventions were undertaken in the late 1950's when the World Health Organization recommended MDA as a tool for malaria control when other methods had failed (reviewed in [32]). Despite successes of MDA in isolated areas [33,34], positive but not always well-documented experiences from China (described in [35]) and a report from Cambodia suggesting that ACT treatment campaigns contributed towards a large reduction in transmission intensity [35], no MDA programme has succeeded in eliminating malaria or leading to a sustainable reduction in the burden of malaria in areas in sub Saharan Africa [32,36].

Despite these sobering results in the past, there is currently a renewed interest in MDA [15,35] because of the previous successes in malaria control [1-6]; highly efficacious anti-malarial drugs with a transmission reducing potential [14-20,37] and new insights in the nature of malaria transmission using sensitive parasite detection methods [27]. Mathematical modelling has shown that malaria transmission is, in theory, susceptible to mass treatment programmes if all subjects within an endemic community could be given anti-malarials that clear parasitaemia during a period of absent or very low vector densities in areas where the intensity of transmission is already low [38,39]. The current manuscript reports on a cluster-randomized trial of MDA with a gametocytocidal drug combination of an ACT combined with primaquine in an area of very low transmission intensity in Tanzania.

## Methods

### Study area and participants

This study was conducted in the area of Lower Moshi (latitude 3°61'-3°68'S; longitude 37°32'-37°38'E), northern Tanzania. The area of Lower Moshi lies on an altitude of ~700 meter between the Maasai savannah and foothills of Mount Kilimanjaro. In 2005, malaria transmission was hypoendemic with an estimated entomologic inoculation rate of 3.4 (95% CI 0.7-9.9) infectious bites per person per year [40]. Mosquitoes are practically absent in the period January-March and more prevalent in March-May and October-November following seasonal rains [40]. Four villages were included in the current study: Kiruani, Majengo, Magadini and Korongo. The total population was 5,094 inhabitants, as determined by a census several weeks before the intervention.

### Consenting procedure and ethical issues

Consent was obtained at three levels. Firstly, village meetings were organized 4-5 months prior to the intervention. During these meetings the study was explained to all village leaders and local ten-cell leaders (representatives of approximately 10 households, *balozi's*) and verbal approval was obtained from these local leaders. The necessity to allow individual households and household members to decide on participation was also explained in order to minimize the binding influence of local authorities. Secondly, verbal household consent was obtained 2-3 months before the intervention during a final census visit in which each household was visited, members were enumerated and their name, gender and age recorded. During this census visit the purpose of the study, its procedures and the practical consequences of participation were explained in detail to all adult household members. Participant information sheets were provided to each head of household and the same information was posted at central places in the village on poster format. Thirdly, individual consent was obtained on the day of the intervention. Prior to drug administration, written consent was obtained from all literate adults, literate household members were asked to sign for consenting illiterate adults. Parents or guardians were asked to consent for children (aged 1-18 years); written consent was also asked for children aged ≥ 10 years. Ethical approval was given by the ethical board of the Tanzanian National Institute of Medical Research (NIMR/HQ/R.8a/Vol. IX/491) and the Tanzanian Food and Drug Authority (CE.57/180/01/8).

### Cluster definition and randomisation

**Figure 1:**
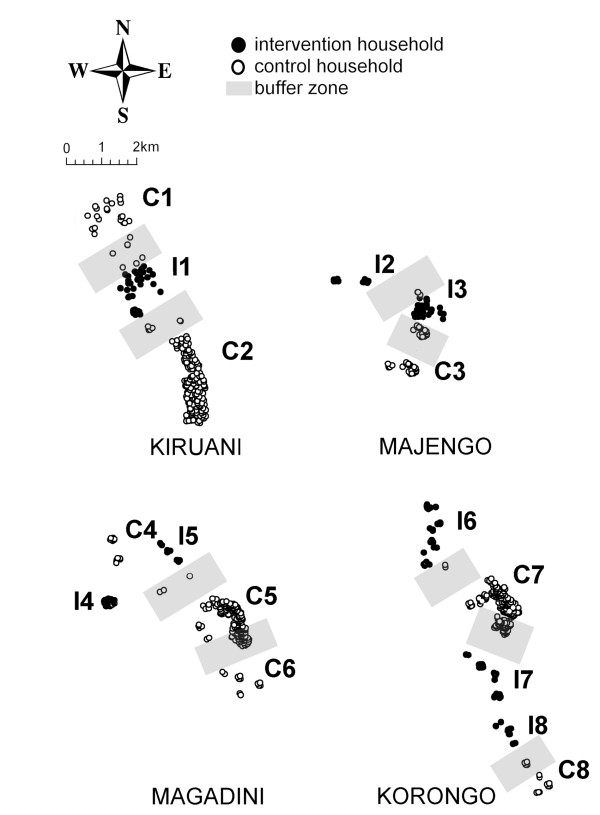
**The study villages of Kiruani, Majengo, Magadini and Korongo**. Each village is presented separately. The north-south arrow relates to the position of households within a village, not to the relative location of villages. Each dot represents a household, dark dots represent intervention households; open dots control households. Buffer zones are depicted in transparent grey. The intervention (I) and control (C) clusters are numbered.

All households and structures in the study area were geo-located using a hand-held global positioning system unit (Trimble GeoExplorer III, California, US). Sixteen geographical clusters were defined using ArcGIS 9.1 (ESRI, Redlands, CA, US), see Figure 1. Where possible, clusters were based on geographically confined groups of households. There was no minimum or maximum population size of clusters but the minimum distance between clusters was set at 1 km, based on the average dispersal range of anophelines in densely populated African settings [41-43]. Clusters were randomized to the intervention or control arm using computer generated randomisation tables using excel (MS Excel, Microsoft Corp., Seattle, US).

Households that were located between clusters (i.e. within 1 km distance from the boundary of intervention and/or control clusters) were considered as buffer zones. Members of these households received the intervention in order to minimize contamination of parasite carriage in the vicinity of intervention clusters, but were not included in the evaluation of the MDA.

### Intervention arms

#### Control arm

Inhabitants of clusters randomized to the control arm received placebo tablets (lactose tablets, Albocin^® ^Glaxo Smith Kline, Brentford, UK) once daily over three days. Placebo tablets were of a different size and colour than the tablets used in the intervention arm.

#### Intervention arm

inhabitants of clusters randomized to the intervention arm received a combination of sulphadoxine-pyrimethamine (25 mg sulphadoxine + 1.25 mg/kg pyrimethamine as single dose on the first day; Fansidar^®^, Roche, Switzerland), artesunate (4 mg/kg/day for three days; Arsumax^®^, Sanofi, France), and primaquine (0.75 mg/kg primaquine as single dose on the third day of treatment in conjunction with the last dose of AS; Tiofarma BV, the Netherlands). Several exceptions were made for safety reasons: i) children below one year of age received no anti-malarial drugs; ii) individuals who had received a full dose of ACT in the two weeks before the intervention received no anti-malarial drugs; iii) pregnant women were not eligible for PQ or AS and received SP (25 mg sulphadoxine + 1.25 mg/kg pyrimethamine as single dose on the first day; Fansidar^®^, Roche, Switzerland) + amodiaquine (10 mg/kg Camoquine^® ^once daily for 3 days; Pfizer, Dakar, Senegal); iv) anaemic individuals (detailed below) received SP+AS only.

### Treatment administration

Seven study teams consisting of one medically-trained individual (medical doctor or medical officer) and one or two field workers were responsible for consenting, screening participants for the safety criteria described above and administering drugs. Several central places were selected in a village, typically the house of the *balozi *or a nearby building. All treatment was given under direct supervision with food to minimize gastro-intestinal complaints. Individuals were treated based on age-groups (Table 1), after average weight for age was determined in cross-sectional surveys that included 2753 individuals as described elsewhere [28,44].

**Table 1:** Drug treatment in the intervention arm by age category

Age	Average weight, median (IQR)	SP	AS	PQ
1-2 y	11.0 (9.5 - 11.6)	250/12.5	25	7.5

3-4 y	12.9 (11.5 - 14.5)	500/25	50	15

5-7 y	16.7 (14.8 - 19.0)	500/25	75	15

8-11 y	23.0 (20.5 - 27.1)	750/37.5	100	22.5

12-14 y	33.7 (28.0 - 38.8)	1000/50	125	22.5

15-16 y	43.3 (38.4 - 54.0)	1000/50	150	30

17-18 y	52.5 (47.9 - 56.5)	1250/67.5	200	37.5

> 18 y	54.0 (46.0 - 61.5)	1500/75	200	45

IQR = interquartile range; SP = sulphadoxine-pyrimethamine; AS = artesunate; PQ = primaquine. SP tablets were given as the nearest complete tablet; AS as the nearest ½ tablet; PQ tablets were given as 7.5 mg or 15 mg tablets or a combination.

Women between 15 and 45 years of age were offered a pregnancy test before treatment was given (SP+AS+PQ for non-pregnant, SP+AQ for pregnant women). If nail bed, palms or conjunctiva were pale, the latter examined with a colour scale [45], people were considered clinically anaemic. In children below 12 years of age, where anaemia was most common [28], haemoglobin was measured by HemoCue photometer [Angelholm, Sweden] and anaemia was defined as an Hb < 8 g/dL. (Clinically) anaemic individuals received SP+AS instead of SP+AS+PQ.

**Figure 2:**
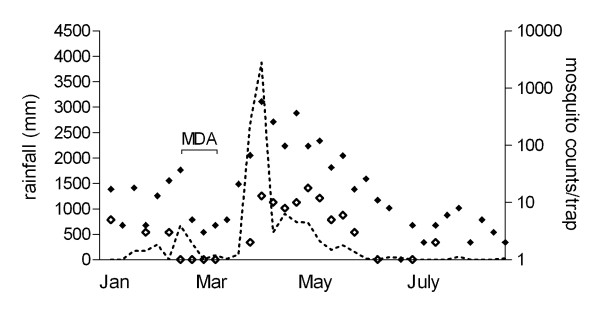
**Seasonality, entomology and timing of the intervention**. Rainfall data is given in the dashed line in mm/week; the average number of female anophelines caught per night in five sentinel houses in Kiruani is represented by solid diamonds; the average number of female anophelines caught per night in ten sentinel houses in Magadini and Korongo is represented by open diamonds. The drug intervention was completed 3-5 weeks before the start of the rains in early April.

The intervention took place in a total period of 16 days, 3-5 weeks before the start of the transmission season (Figure 2). All *balozi's *(representatives of ~10 households) were involved in the monitoring of movement of people in the study area. If people newly arrived in the months following the intervention, *balozi's *contacted the study team that promptly administered the treatment according to household allocation. A random selection of children below 12 years of age were monitored actively for adverse events [44]; for the rest of the population adverse events were monitored passively. The likelihood that adverse events were related to the intervention was assessed based on known previously reported side-effects of the study drugs (including a-specific allergic reactions) and the timing of symptoms in relation to drug administration. Conventional definitions of severity (adverse events, serious adverse events) and relatedness (unrelated, unlikely, possible, probable and definite) were used.

### Objective

To determine the impact of a single round of mass drug administration with a gametocytocidal drug combination on malaria transmission in Lower Moshi, an area of low and seasonal malaria transmission.

### Outcome measures

Outcome measures were the impact of MDA on i) malaria morbidity by active and passive case detection; ii) asexual parasite prevalence and density by microscopy, rapid diagnostic test and molecular detection methods; iii) gametocyte prevalence and density by microscopy and molecular detection methods; iv) transmission intensity quantified by the entomologic inoculation rate.

### Malaria morbidity by active and passive case detection

The impact of the intervention on malaria morbidity was assessed by active and passive case detection. Three health facilities in the study area were involved in passive case detection. Each household was provided with a household identification card that contained the names of household members, information on their sub-village, village leader and household number that allowed geo-locating the households. With this household identification card, all household members were entitled free access to treatment at the health facilities. Upon arrival at the health facility, household number, name, age and gender were recorded by the responsible medical doctor or clinical officer. Temperature was measured and a slide and rapid diagnostic test (RDT) prepared. Diagnosis was based on the RDT result and parasitaemic individuals with (history of) fever were treated with artemether-lumefantrine according to national guidelines. For active case detection, 150 children (aged 1-10 years) from intervention clusters and 150 children (aged 1-10 years) from placebo clusters were randomly selected to be followed-up for six months. Caretakers of these children were provided with a simple symptom diary and asked to record the daily occurrence of fever, headache and body pains. Every fourteen days a trained fieldworker visited the child, examined their symptom diary and measured their temperature. In case of (history of) fever, an RDT was used to determine the presence of parasites. Parasitaemic children and all children with current fever were transported to the nearest health facility for clinical care and, in case of a positive RDT, confirmation of parasitaemia by microscopy.

### Parasite carriage in cross-sectional surveys

A cross-sectional survey was conducted in January-February, several weeks before the intervention. Four cross-sectional surveys were conducted to evaluate the impact of the intervention in April-July. On each occasion, 50 individuals from each cluster were individually selected from computer generated random tables and invited to a central point in one of the four sub-villages. A short questionnaire was administered and participants were asked to provide a blood sample for i) rapid diagnostic test (RDT, Paracheck^® ^Orchid Biomedical Systems, Goa, India); ii) blood slide; iii) serology from filter paper (3 MM Whatman, Maidstone, UK); iv) extraction of nucleic acids from filter paper (903 Protein Saver Card, Whatman, Maidstone, UK); v) hemoglobin concentration by HemoCue photometer (HemoCue, Angelholm, Sweden). Diagnosis was based on the RDT; those positive by RDT with (a history of) fever were treated with artemether-lumefantrine (Coartem^®^, Novartis, France) according to national guidelines.

Slides were Giemsa-stained and read by two independent microscopists, each examining 100 high power fields before declaring a slide negative. If parasites were detected, these were enumerated against 200 white blood cells and converted to parasite density/μL assuming 8,000 white blood cells/μL of blood.

Exactly 50 μL of blood was blotted onto filter paper (903 Protein Saver Card, Whatman Maidstone, UK), dried overnight and stored with silica gel at -20°C. Nucleic acids were extracted following the procedure of Boom *et al *[46], as described for filter paper by Mens *et al *[47]. RNA samples were tested by quantitative nucleic acid sequence based amplification (QT-NASBA) on a NucliSens EasyQ analyzer (bioMérieux, Boxtel, the Netherland) for 18S rRNA parasite detection as described elsewhere with a sensitivity of ~20 parasites/mL [48]; 18S rRNA is present in all asexual parasites and gametocytes. The time to positivity, i.e., the time point during amplification at which the number of target amplicons detected exceeded the mean for three negative controls plus 20 standard deviations, was calculated. Those positive in the 18S QT-NASBA, were scheduled for testing in the gametocyte specific Pfs25 QT-NASBA, that has a detection limit of 20-100 gametocytes/mL [48]. For an initial analysis, 150 samples were randomly selected from the pre-intervention survey (75 intervention and 75 control arm) and 100 samples per follow-up cross sectional survey (50 intervention and 50 control arm).

### Transmission intensity by entomological inoculation rate

Mosquitoes were collected in the period October 2007 - July 2008 in the sub-villages Korongo, Magadini and Kiruani. Ten households were selected from each sub-village, 5 from the intervention and 5 from the control clusters for each village. Households were selected to be representative of the typical housing structures in the villages, mud wall and corrugated iron roof. Mosquitoes were caught with a standard Centre for Disease Control light traps (CDC, Atlanta, USA). Traps were hung at the end of an occupied bed with a bed net that was newly provided by the investigators. Traps were set for 12 hours, from 7 pm to 7 am [49]. In the morning, traps were then collected and mosquito species determined and counted. Male anophelines and Culicines were discarded. Female *Anopheles *mosquitoes were stored on silica gel for circumsporozoite protein (CSP) ELISA as described by Wirtz *et al *[50]. Samples were prepared individually and assayed in batches of five with positive batches re-assayed as single mosquitoes. Insectary-reared unfed female *Anopheles *mosquitoes were used as negative controls with the kit supplied CSP antigen as positive control. Samples were read by eye and on an ELISA plate reader at 495 nm. The Entomologic Inoculation Rate was calculated per person per year [51].

### Sample size considerations

The number of clusters needed for the evaluation was based on the expected baseline parasite prevalence by 18S QT-NASBA of 30% [28] and a 1:1 allocation over intervention and control arms. A pilot study indicated that parasites were cleared completely within 14 days [37] and it was assumed that MDA would reduce parasite carriage in the intervention clusters to below 10%. Including 50 individuals per cluster in the post-intervention cross-sectional surveys and assuming a conservative estimate of k (0.5 coefficient of variation of true proportions between clusters within each group), 8 clusters per intervention arm were needed (Z_α/2 _= 1.96; Z_β _= 0.84). To be able to detect temporal variation in efficacy, the study aimed to collect 50 observations per cluster in the early transmission season (April-May) and 50 in the late transmission season (June-July). The number of children included in active case detection was based on an expected malaria incidence of 0.5 malaria episode/child/year in the control arm and a tenfold lower incidence in the intervention arm. Enrolling 150 children per treatment arm for six months would result in 900 person-months per arm, giving 80% power to detect the specified outcomes after, adjusting for correlation between observations from the same cluster.

### Statistical analysis

Parasite prevalence by microscopy and molecular methods was compared between study arms using Generalized Estimating Equations (GEE) to account for correlations between observations from the same cluster and the same survey. Poisson regression was used for the comparison of malaria incidence between the intervention and control arm, using GEE to account for correlations among children residing in the same cluster.

## Results

### Participant flow

The total population of the study area comprised 5,094 individuals living in 1,253 households. Sixteen clusters were generated, consisting of 55 to 737 individuals.

**Figure 3:**
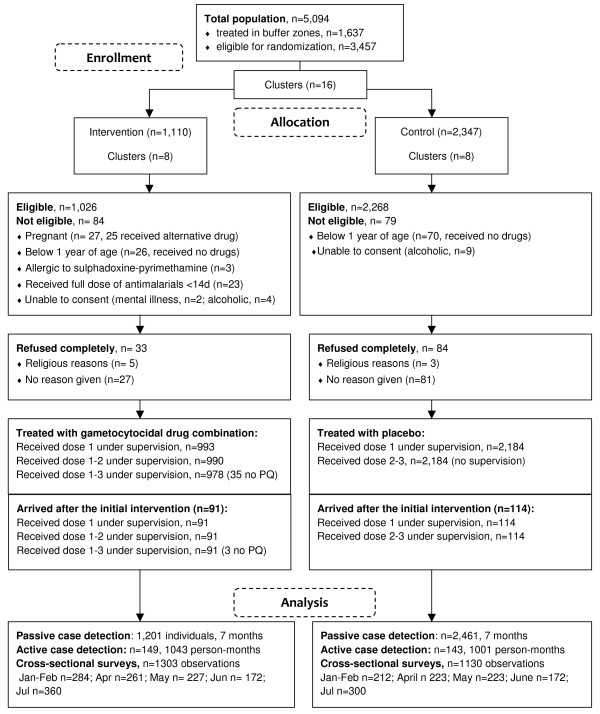
**Participant flow chart**.

Eight clusters with a total of 1,110 individuals were randomized to receive the intervention, eight clusters with a total of 2,347 individuals were randomized to receive placebo (Table 2). The three largest clusters with 737, 649 and 525 individuals were all randomized to the placebo arm, explaining the difference in population size between the two treatment arms. Of those eligible 96.8% (993/1,026) received at least one dose of the gametocytocidal drug combination, 96.5% (990/1,026) received two doses and 95.3% (978/1,026) received three doses although 35 of these were excluded from PQ treatment because of a measured Hb < 8 g/dL (Figure 3). Twenty-seven women were pregnant; 25 of them received alternative malaria medication with SP+AQ.

**Table 2:** Baseline data

	Intervention (8 clusters)	Placebo (n = 8 clusters)
Present at the time of the intervention	1,110	2,347
Arrived in the study area after the intervention	91	114
Proportion female, % (95% CI)	50.0 (47.0 - 53.1)	49.2 (47.2 - 51.1)
Age, median (IQR)	12 (5 - 26)	16 (6-32)
Parasite positive by rapid diagnostic test, % (95% CI)	0.4 (0.0 - 2.0)	1.0 (0.1 - 3.4)
Parasite positive by microscopy, % (95% CI)	0.0 (0.0 - 1.3)	0.0 (0.0 - 1.8)

A total of 91 individuals (25 children < 15 years, 3 children < 1 year) arrived in an intervention area and 114 individuals (41 children < 15 years; 6 children < 1 year) arrived in a placebo area after the intervention took place (Table 2); all agreed to take the drugs their area of residence was allocated to within two weeks of arrival and under supervision. 94.3% (1,132/1,201) of the total population of the intervention clusters, including newcomers and pregnant women (receiving alternative medication) received at least one dose and 93.0% (1,117/1,201) of these received a complete dose of efficacious anti-malarial drugs in the month preceding the start of the transmission season or immediately upon arrival in the area.

### Adverse events

One individual was diagnosed to have a severe skin reaction in the week following the intervention. On review of the study physician this was assessed to be possibly drug-related. A second individual presented with skin hyper-pigmentation on the face; this was judged to be non-related to drug exposure. Both individuals were treated with steroid and closely observed until symptoms disappeared. Haemolytic effects of the intervention were described in detail elsewhere [44]. Briefly, statistically significant but asymptomatic and transient haemolysis was observed in children who were specifically monitored for this side effect. Haemolysis was most pronounced in G6PD deficient individuals [44].

### The impact of MDA on malaria morbidity

Passive case detection was carried out in the period February-July, during which 405 individuals attended the clinic with fever and suspected malaria. All individuals were screened for malaria parasites by rapid diagnostic test and microscopy. Two malaria cases were confirmed by microscopy and RDT during passive case detection: a 65-year old man with a parasite density of 4,951 parasites/μL and a 32-year old woman with an asexual parasite density of 800 parasites/μL and 16 gametocytes/μL. Both individuals resided in control clusters. Active case detection was done for 149 children below 10 years of age residing in intervention clusters and 143 children of the same age from control clusters. One child from an intervention cluster was identified with reported fever and a parasite density of 240 parasites/μL. None of the malaria cases reported travelling in the month prior to their symptoms.

### The impact of MDA on malaria parasite prevalence

Parasites were detected by microscopy in 1.9% (95% CI 1.4-2.5) of the individuals by microscopy and 32.5% (95% CI 28.2-37.0) of the individuals by QT-NASBA in 2005 [28] (Table 3). In the pre-intervention cross-sectional survey, parasites were detected in 0.0% (95% CI 0.0-1.0) of the individuals by microscopy and 2.8% (95% CI 0.8-6.9) by QT-NASBA. In all post-intervention surveys, parasites were not detected in any of the examined slides (0/2434) and in only six of the examined blood spots (1.1%, 6/537) examined by QT-NASBA. After this initial evaluation, the laborious and costly parasite detection by QT-NASBA was stopped because it was evident that the study was underpowered to detect differences in parasite carriage between intervention and control clusters.

**Table 3:** Parasite carriage before and after the intervention

			Asexual parasite carriage		Gametocyte carriage	
			microscope	QT-NASBA	microscope	QT-NASBA
2005			1.9 (1.4-2.5)	32.5 (28.2-37.0)	0.4 (0.2-0.7)	15.0 (11.9-18.7)
2008	Pre-intervention	Intervention	0.0 (0-2.1)	2.6 (0.3 - 9.2)	0.0 (0-2.1)	ND
		Control	0.0 (0-1.7)	2.9 (0.04 - 10.1)	0.0 (0-1.7)	ND
	Evaluation I	Intervention	0.0 (0-1.8)	0.0 (0.0 - 6.5)	0.0 (0-1.8)	ND
		Control	0.0 (0-1.6)	0.0 (0.0 - 7.2)	0.0 (0-1.6)	ND
	Evaluation II-IV	Intervention	0.0 (0-0.7)	0.0 (0.0 - 2.4	0.0 (0-0.7)	ND
		Control	0.0 (0-0.5)	1.4 (0.2 - 5.1)	0.0 (0-0.5)	ND

Asexual parasite carriage was determined by microscopy, reading 100 microscopic fields, and 18S rRNA-based QT-NASBA that detects all parasite stages at an estimated sensitivity of 20 parasites/mL. Gametocytes were detected by microscopy, separately reading 100 microscopic fields, and *Pfs25 *mRNA based QT-NASBA that is gametocyte specific and has a sensitivity of 20-100 gametocytes/mL. ND = not done

### The impact of MDA on malaria transmission intensity

A total of 14,586 mosquitoes were caught in 491 trapping nights in the intervention clusters and 10,944 during 478 trapping nights in the control clusters. The seasonality of mosquito exposure is presented in Figure 2. Only one mosquito from a control cluster in Kiruani (May) was found to be infected with sporozoites (0.004%, 1/24,528), resulting in an estimated EIR for the study area of 0.63 infectious bites per person per year.

## Discussion

The current manuscript describes the first MDA study on the African continent since 1999 [36]. A coverage of 94% with at least one dose of anti-malarials was achieved; 93% with the full per protocol dose of three drugs given over three days. Despite this high participation rate, no impact of MDA on malaria transmission was observed. Parasite prevalence rates before and after the intervention, by microscopy and PCR, were too low to allow conclusions on the impact of the intervention.

The success of MDA depends on its capacity to reduce the prevalence of pathogens below a critical threshold level where re-emergence is unlikely and control can be maintained [52]. For malaria, this threshold prevalence is very low; the basic reproductive number (R_0_), the number of secondary episodes arising from a single infectious human case in a fully susceptible population, can be well over 100 if malaria vectors are present [53]. The chance of achieving this threshold is highest when parasites are cleared from the human population towards the end of the dry season when very few mosquitoes are present [38]. The MDA intervention was completed three weeks before the increase in mosquito numbers following the seasonal rains and used a gametocytocidal drug combination of SP+AS+PQ that clears gametocytes in an average of 6.3 days; significantly quicker than after ACTs alone [54]. Despite the demanding three-day dosing scheme and logistical challenges to exclude pregnant women from treatment with AS and PQ [55,56], our coverage was high compared to that of previous malaria MDAs. Of the total population living in intervention clusters, 94% received at least one dose and 93% received a complete dose of efficacious anti-malarial drugs in the month preceding the start of the transmission season or immediately upon arrival in the area. In The Gambia in 1999, a coverage rate of 73% was achieved after one and of 88% after two rounds of drug administration. The drug combination given was SP plus a single dose of AS, given on a single day [36,57]. Coverage of other reported MDA campaigns in Uganda (50%) [34], Nicaragua (70%) [58] and Garki, Nigeria (85%) [59] were lower than that in the current setting. The successful malaria eradication campaign on Aneityum, Vanuatu, used 9 rounds of MDA in a population of 718 individuals with participation rates of 79-92% for each round [33]. The maximum number of tablets that had to be taken by adults on Aneityum was 13 on a single day, compared to 7 per day on the first two days and 10 on the third day in the current study. Although this indicates that a complicated dosing scheme and high tablet number do not preclude high participation rates, the number of tablets was mentioned as burdensome by some participants (Shekalaghe, unpublished data) [33,60].

The intervention strategy that involved local village leaders and village representatives as part of the intervention teams is likely to have contributed to the high level of community participation [60]. The stepwise consent procedure and repeated village meetings gave inhabitants of the study area sufficient time to consider participation and opportunities to raise questions. A study on community perceptions of MDA in The Gambia concluded that knowledge about malaria was related to a higher individual participation rate and that people who had discussed the study with other villagers and those who understood the necessity for a high participation rate for MDA to work were also more likely to participate [57]. During community meetings the necessity for asymptomatic individuals to participate in order to achieve community benefits was strongly emphasized, although no attempt was made to quantify community understanding. The approach to give local leaders a central role in explaining the study purposes, drug administration and monitoring migration will also have influenced the response rate. Several opinion makers in The Gambia were antagonistic to the intervention and reduced participation rates [57]; the current study received full participation of all 62 *balozi's *in the study area.

Despite its high coverage, no impact of MDA on the transmission in the area was observed. The study area was selected for its very low transmission intensity and marked seasonality with almost complete absence of vectors for several months per year [40]. The site selection and trial design were based on the assumption of a sub-microscopic parasite carriage of ≥ 30% [28], that would maintain malaria transmission in the area [31] and would form a suitable primary outcome measure for the intervention. Contrary to these expectations, malaria had all but disappeared from the area prior to the MDA intervention during which no major additional malaria specific control efforts were undertaken. Considerable reductions in transmission intensity without evident underlying causes have been reported across northern Tanzania [5,61,62]. Reported use of insecticide treated net (ITN) in this period increased from 25.1% (490/1952) in 2004 [28] to 36.1% (879/2434) in 2007. If these numbers reflected a change in effective ITN use, this could have resulted in a substantial reduction in parasite prevalence in the study area of low endemicity [63] although coverage remains too low to explain an almost complete elimination of malaria in the area [64,65]. Presumptive treatment with ACT could also have contributed to the decline in transmission intensity. Although treatment prescribing behaviour in the area was not quantified, presumptive treatment with artemether-lumefantrine, introduced as first-line treatment in 2007, was common practice and will have provided parasitaemic and non-parasitaemic individuals with a curative dose and effective prophylaxis for several weeks [18,66]. Rainfall was unreliable in the period preceding the MDA intervention; being 34% of the preceding 10-year average in 2004, 35% in 2005, 116% in 2006 and 66% in 2007. A single treatment campaign for trachoma with azithromycin was undertaken by a non-governmental organisation with an unknown coverage level. Azithromycin has some anti-malarial activity at doses given for trachoma [67]. These components may all have contributed to the fall in transmission intensity observed. The findings from this study also illustrate the limited understanding on the importance of sub-microscopic parasite densities for maintaining stable malaria transmission. In 2005, a large-scale survey was conducted in the study area and detected *P. falciparum *in 32.5% of the general population by PCR compared to 1.9% by microscopy [28]. The large proportion of sub-microscopic parasite carriers is typical for areas of low endemicity; in areas where the microscopic parasite prevalence is below 10%, microscopy on average detects just under a quarter of all parasite carriers [30]. Although low-density infections can be highly efficient in eliciting protective immunity in experimental infections [68], their dynamics are poorly understood in endemic settings. Harris and colleagues observed that in a low endemic area on the Solomon islands, parasites were carried at microscopically detectable densities by 2.2% of the population of whom > 85% were afebrile. The majority of parasite carriers in all age groups had parasites at densities below 500 parasites/μL and parasite prevalence increased almost four-fold when PCR was used for detection [69]. Low parasitaemia and frequent asymptomatic parasite carriage was also reported for an area of very low endemicity in Vanuatu where parasite prevalence by microscopy was 2-3% and > 80% of the parasite carriers harboured parasites asymptomatically and at a densities below 500 parasites/μL or below the microscopic threshold for detection [70]. It is currently unknown how individuals with limited cumulative exposure to malaria manage to control malaria at low parasite densities or how long sub-microscopic infections persist. The average duration of malaria infections has been estimated at 210 days in area of intense malaria transmission [71] where most infections are microscopically detectable [30]. The duration and gametocytaemic period of sub-microscopic infections, that dominate in low endemic areas, are currently unknown. These factors are crucial in determining the relative contribution of sub-microscopic infections, that are sufficient to infect mosquitoes [26,29], to malaria transmission in a population.

## Conclusions

This manuscript reports on the first MDA study in Africa in more than a decade and achieved an unsurpassed participation rate. Malaria transmission had practically vanished prior to the intervention, with no microscopic parasite carriers in ~2,400 slides and < 2% parasite carriage in the subset of samples that were tested for *P. falciparum *by QT-NASBA. Molecular parasite detection methods form a sensitive alternative to microscopy in low endemic settings but some important epidemiological and clinical aspects of sub-microscopic parasite carriage are currently poorly understood and need elucidation. The current study indicates that it is complicated to determine the impact of MDA on malaria transmission as stand-alone intervention; this requires a study area of very low and seasonal yet stable malaria transmission intensity. Future studies that aim to determine the impact of MDA in Africa will need to select areas with a higher pre-intervention level of malaria transmission intensity than the site in northern Tanzania where this study was conducted. Considering the current uncertainties about the stability and longevity of sub-microscopic parasite carriage, it may be advisable to base power calculations for these future MDA studies on microscopic parasite carriage in the population.

## Competing interests

The authors declare that they have no competing interests.

## Authors' contributions

SAS contributed to data acquisition, data analysis interpretation, and to the preparation of the manuscript. SVDB, RTB and WVDB contributed to data acquisition in the field, data analyses and interpretation. CM, SS, AM and FM contributed to data acquisition in the field. KT and RH contributed to molecular assays. LO contributed to data interpretation. RG contributed to the study design and data acquisition in the field and data interpretation. CD contributed to the study design and data acquisition in the field, data interpretation and manuscript preparation. RS contributed to the study design, the interpretation of the data and manuscript preparation. TB contributed to the study design, data acquisition in the field, data analysis, data interpretation and manuscript preparation. All authors read and approved the final manuscript.
